# Robot-Assisted Right Hemicolectomy for Ascending Colon Cancer in a Kidney Transplant Recipient: A Case Report

**DOI:** 10.7759/cureus.109275

**Published:** 2026-05-20

**Authors:** Yasuhiro Takahashi, Kosuke Hiramatsu, Jumpei Kashiwagi, Daisuke Tomita, Hiroya Kuroyanagi

**Affiliations:** 1 Department of Gastroenterological Surgery, Toranomon Hospital, Tokyo, JPN

**Keywords:** colon cancer, kidney transplantation, port placement, renal allograft, right hemicolectomy, robot-assisted surgery

## Abstract

During colorectal surgery, the renal allograft and transplanted ureter may constrain port placement and instrument movement; therefore, kidney transplant recipients require careful planning. Here, we report a case of robot-assisted right hemicolectomy for ascending colon cancer adjacent to an ipsilateral renal allograft. A 62-year-old woman underwent deceased-donor kidney transplantation 16 years prior, with a renal allograft placed in the right iliac fossa. Colonoscopy revealed a near-circumferential lesion in the ascending colon, and biopsy revealed a well- to moderately differentiated tubular adenocarcinoma with focal mucinous components. Non-contrast computed tomography (CT) showed that the tumor was adjacent to the renal allograft, with no lymph nodes or distant metastases. Preoperative CT was used to assess the anatomical relationships between the tumor, renal allograft, and transplanted ureter. Robot-assisted right hemicolectomy was performed using the Hugo™ RAS system (Medtronic plc, Minneapolis, MN, USA). Arm 1 port was shifted medially to avoid the renal allograft, and robotic arm clearance and instrument mobility were confirmed before dissection. The procedure was completed without any conversion. The operative time was three hours and 24 minutes, and the estimated blood loss was less than 10 mL. The final pathology revealed pT3N0M0 stage IIA disease with negative margins and no lymph node metastases. No postoperative complications, graft rejection, or graft dysfunction were observed. This case suggests that robot-assisted right hemicolectomy may be feasible in selected kidney transplant recipients when patient-specific anatomy is assessed preoperatively, port placement is individualized, and robotic arm clearance and instrument mobility are confirmed.

## Introduction

In kidney transplant recipients, the cumulative risk of malignancy is known to increase over time in the setting of long-term immunosuppressive therapy and prolonged post-transplant survival [1]. Colorectal cancer is one such malignancy, and kidney transplant recipients have been reported to have a higher risk of developing advanced colorectal neoplastic lesions and colorectal cancer than the general population [2,3]. Surgical resection remains the cornerstone of treatment for resectable colorectal cancer [4]; however, in kidney transplant recipients, perioperative adverse events such as infectious complications, wound-related complications, and acute kidney injury require particular attention [5,6]. Additionally, the risk of intraoperative injury related to the presence of renal allografts and transplanted ureters must be considered.

In this context, minimally invasive approaches, including laparoscopy and robot-assisted surgery, may offer advantages such as magnified visualization, improved anatomical recognition, faster postoperative recovery, and reduced wound-related complications [7,8]. However, their applicability depends on patient-specific anatomical factors, including the location of the renal allograft and the course of the transplanted ureter.

Although minimally invasive colorectal resection, including laparoscopic and robotic approaches, has been described in kidney transplant recipients, detailed descriptions of robot-assisted right hemicolectomy for right-sided colon cancer located adjacent to an ipsilateral renal allograft are limited [9-11]. In such cases, the operative strategy must be individualized to accommodate tumor location and transplant-specific anatomy, including the renal allograft and transplanted ureter, while ensuring safe port placement and adequate robotic access. Here, we report a case of robot-assisted right hemicolectomy for ascending colon cancer adjacent to an ipsilateral renal allograft using the Hugo™ RAS system (Medtronic plc, Minneapolis, MN, USA), focusing on preoperative anatomical assessment, individualized port placement, and confirmation of robotic-arm clearance before dissection.

## Case presentation

A 62-year-old Japanese woman with membranoproliferative glomerulonephritis as her primary renal disease had been on maintenance hemodialysis since the age of 25 years. At 46 years of age, she underwent kidney transplantation from a deceased donor after circulatory death, with placement of a renal allograft in the right iliac fossa. The patient had no history of rejection.

**Figure 1:**
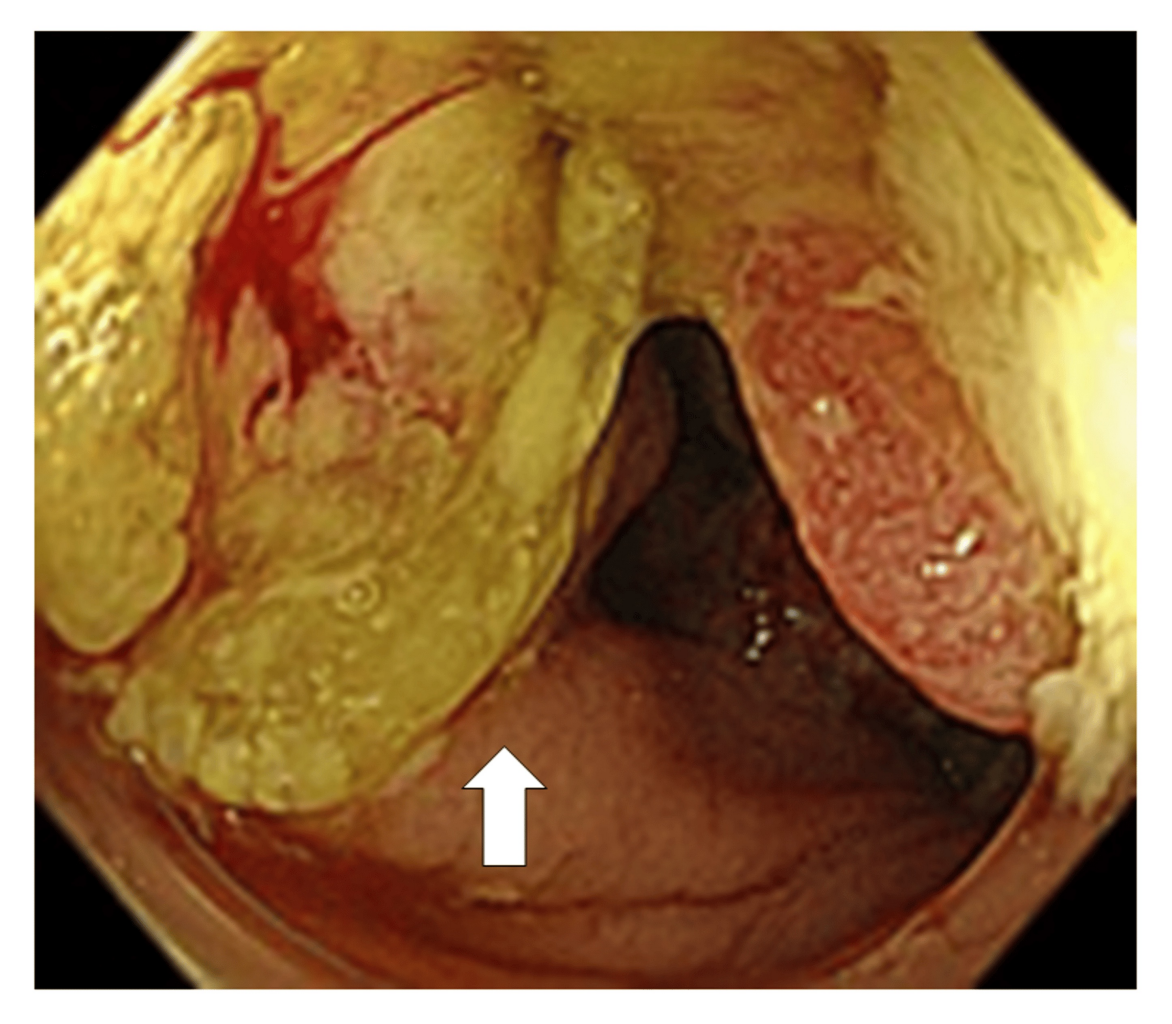
Colonoscopy findings Colonoscopy revealed a near-circumferential lesion in the ascending colon (arrow).

Sixteen years after the kidney transplantation, a fecal occult blood test was positive during a routine health checkup, and subsequent colonoscopy revealed a near-circumferential ulceroinfiltrative lesion in the ascending colon (Figure 1).

**Figure 2:**
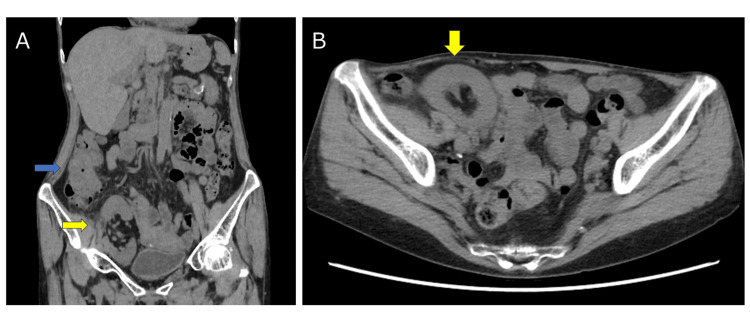
Computed tomography (CT) findings Non-contrast CT showed an ascending colon tumor (blue arrow) and a renal allograft in the right iliac fossa (yellow arrows). (A) Coronal view; (B) Axial view

A biopsy revealed a well- to moderately differentiated tubular adenocarcinoma with focal mucinous components. Non-contrast computed tomography (CT) revealed an irregular mass in the ascending colon adjacent to the renal allograft in the right iliac fossa (Figures 2A, 2B).

The expected course of the transplanted ureter was estimated from the renal pelvis of the allograft toward the bladder in the right hemipelvis. There was no evidence of lymph node or distant metastases.

The patient was referred to our department for further surgical treatment. At the time of referral, the patient’s immunosuppressive regimen consisted of tacrolimus (1.5 mg/day), everolimus (1 mg/day), and methylprednisolone (2 mg/day). Physical examination, including an abdominal examination, was unremarkable. Preoperative laboratory findings are summarized in Table 1.

**Table 1:** Preoperative laboratory findings

Parameter	Value	Reference Range
White blood cell count	11,600/μL	3,300–8,600/μL
C-reactive protein	1.49 mg/dL	≤0.14 mg/dL
Serum creatinine	1.48 mg/dL	0.46–0.79 mg/dL
Carcinoembryonic antigen	3.5 ng/mL	≤5.0 ng/mL
Carbohydrate antigen 19-9	9 U/mL	≤37 U/mL

The results showed a mild inflammatory response and an elevated serum creatinine level, whereas tumor marker levels were within the reference ranges. As the tumor was ipsilateral and adjacent to the renal allograft, securing an adequate operative field and avoiding instrument interference were considered essential when planning a minimally invasive approach. Preoperative assessment of the renal allograft location and the expected course of the transplanted ureter guided port placement away from the right iliac fossa. This assessment also helped identify the area near the expected ureteral course in which deep caudal dissection should be avoided. Robot-assisted surgery was planned with conversion to open surgery as a backup option, if necessary.

**Figure 3:**
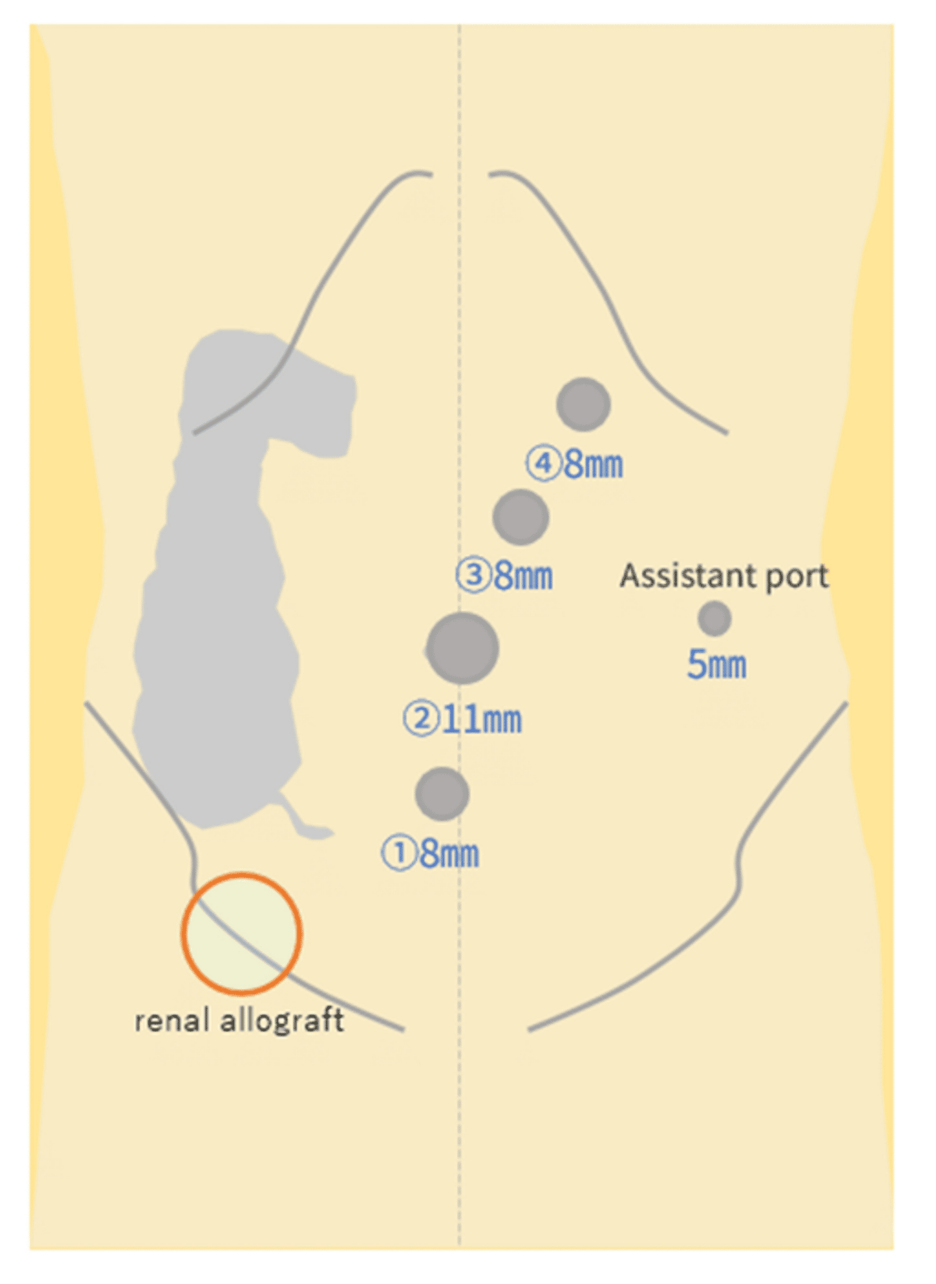
Modified port placement for robot-assisted right hemicolectomy Schematic illustration of modified port placement using the Hugo™ RAS system. The Arm 1 port was shifted 2 cm medially from the usual Arm 1 port site and placed near the midline to avoid the renal allograft in the right iliac fossa. A 5-mm assistant port was placed in the left lateral abdomen. Port placement schematic adapted with permission from a generic template provided by Covidien AG (Mansfield, MA, USA, now part of Medtronic plc, Dublin, Ireland), and modified by the authors using Microsoft PowerPoint (Microsoft Corporation, Redmond, WA, USA) to incorporate case-specific anatomical and operative details based on preoperative CT and intraoperative findings.

Robot-assisted right hemicolectomy was performed using the Hugo™ RAS system under general anesthesia with epidural analgesia. The patient was placed in the lithotomy position. A camera port was inserted through the umbilical incision using an open technique. The pneumoperitoneum was maintained at 8 mmHg. Intra-abdominal inspection revealed no liver metastases or peritoneal dissemination. The tumor was located in the middle of the ascending colon. A renal allograft was confirmed in the right iliac fossa. The robotic ports were placed according to the modified port configuration shown in Figure 3.

**Figure 4:**
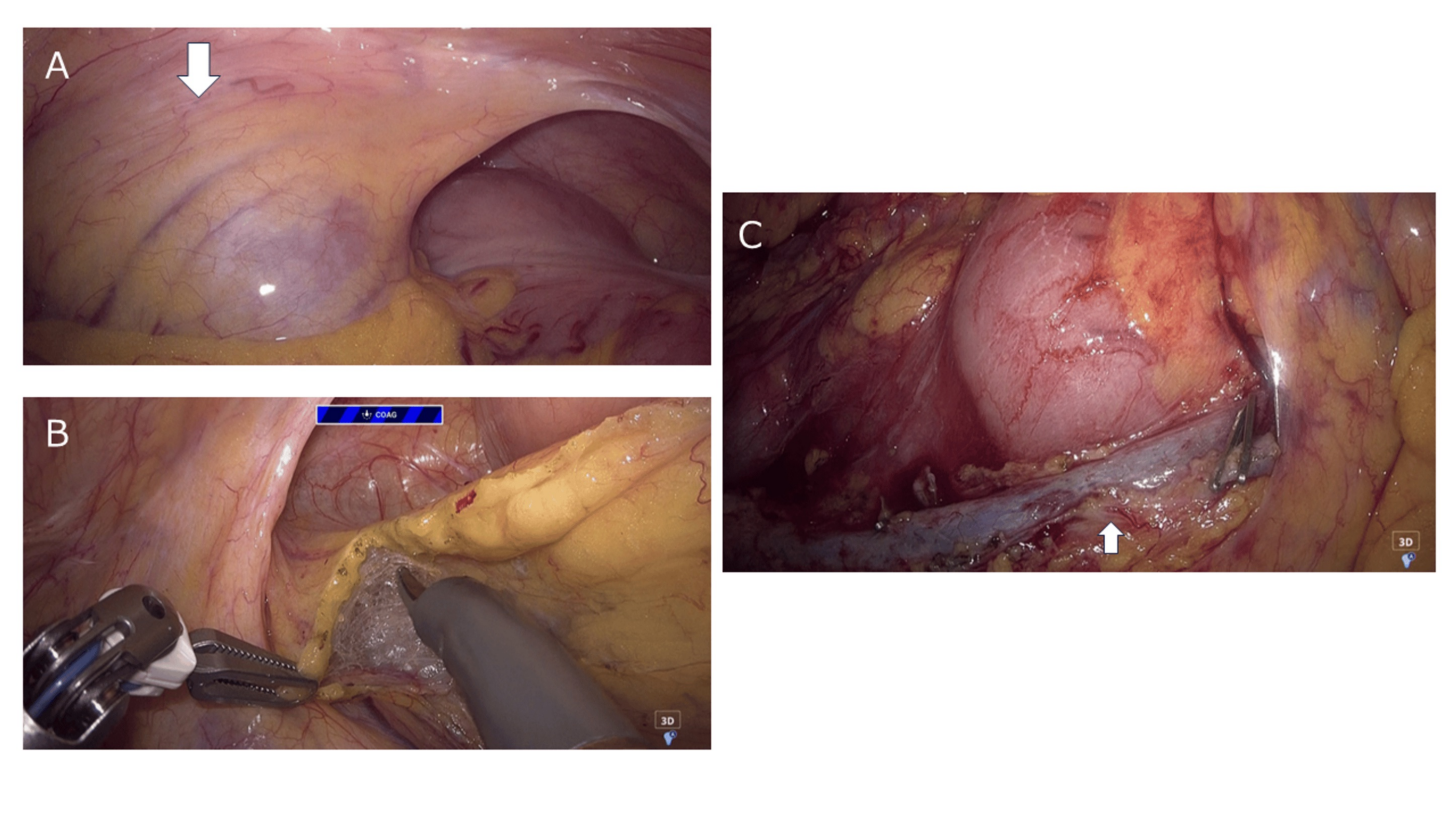
Intraoperative findings (A) The renal allograft was located in the right iliac fossa (arrow). (B) Caudal and lateral mobilization of the ileocecal region was performed while avoiding direct manipulation of the renal allograft region. (C) The operative field after central lymphadenectomy along the superior mesenteric vein (arrow).

The Arm 1 port was shifted 2 cm medially from its usual position and placed near the midline to avoid the renal allograft. The intraoperative location of the renal allograft is shown in Figure 4A.

A 5-mm assistant port was placed in the left lateral aspect of the abdomen to provide countertraction during right colonic mobilization while minimizing manipulation near the renal allograft and pressure on the graft. The docking and tilt angles were adjusted to ensure adequate robotic-arm clearance and instrument mobility. The docking angles, tilt angles, and instruments used with the Hugo™ RAS system are summarized in Table 2.

**Table 2:** Hugo™ RAS System settings and instruments

Robotic Arm	Role	Instrument	Docking Angle	Tilt Angle
Arm 1	Left-hand instrument	Bipolar fenestrated grasper	220°	−25°
Arm 2	Camera	30° endoscope	255°	−30°
Arm 3	Right-hand instrument	Monopolar curved scissors	285°	−45°
Arm 4	Auxiliary grasper	Double-fenestrated grasper	310°	−20°

After docking, we confirmed adequate robotic-arm clearance and instrument range of motion before initiating the dissection. No external robotic-arm collisions or intra-abdominal limitations in instrument movement were observed. Therefore, the procedure was continued robotically. As the Arm 1 instrument was used near the renal allograft during ileocecal mobilization, insertion and exchange through the Arm 1 port were performed under endoscopic visualization. During this step, the operating table was placed in the 10°-15° Trendelenburg position. The wrist articulation of the Arm 1 instrument was used to obtain an appropriate traction angle while avoiding undue pressure on the renal allograft. During the lateral mobilization of the cecum and ascending colon, care was taken to avoid excessive caudal or lateral dissection, which could bring the operative plane close to the renal allograft and the expected course of the transplanted ureter. Therefore, dissection was confined to the mesocolic plane, and direct manipulation around the renal allograft was avoided (Figure 4B). The transplanted ureter was not exposed or manipulated intraoperatively. Instead, unnecessary deep caudal dissection near the expected ureteral course was avoided. As the dissection proceeded cranially and away from the right iliac fossa, the risk of inadvertent contact with the renal allograft decreased. During central lymphadenectomy along the superior mesenteric vein, the operating table was returned from the Trendelenburg position and maintained with the patient’s right side elevated by 5°. The ileocolic and right colic vessels were divided at their origins. Complete mesocolic excision with central lymphadenectomy was achieved, despite the modified port configuration (Figure 4C). After completion of robotic dissection, the umbilical incision was extended for specimen extraction, and an extracorporeal functional end-to-end ileocolic anastomosis was performed. The operative time was 3 h 24 min, and the estimated blood loss was <10 mL.

**Figure 5:**
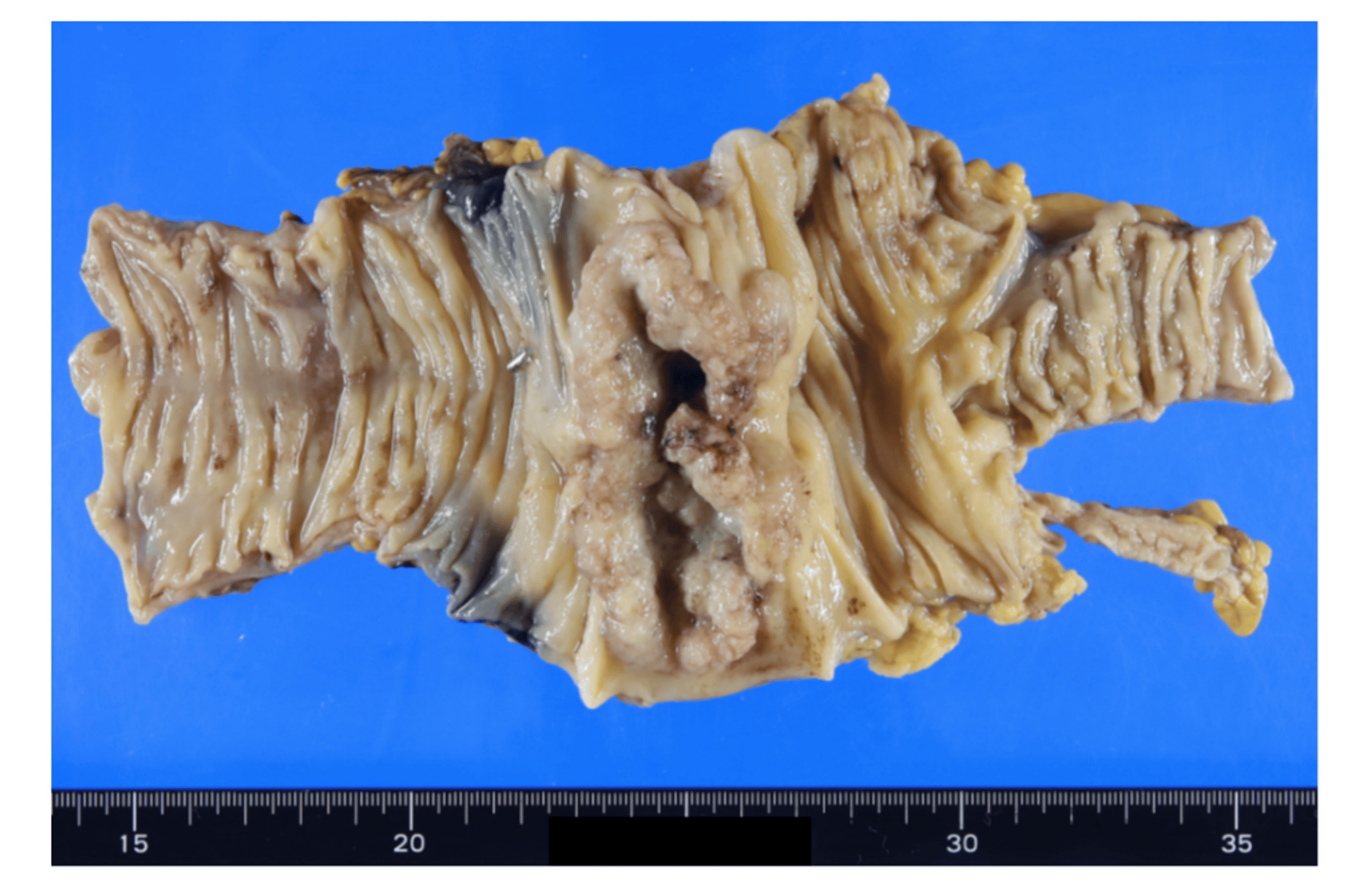
Gross specimen The resected specimen showed a near-circumferential tumor measuring 85 × 43 mm in the ascending colon.

The resected specimen revealed a near-circumferential tumor measuring 85 × 43 mm in the ascending colon (Figure 5).

**Figure 6:**
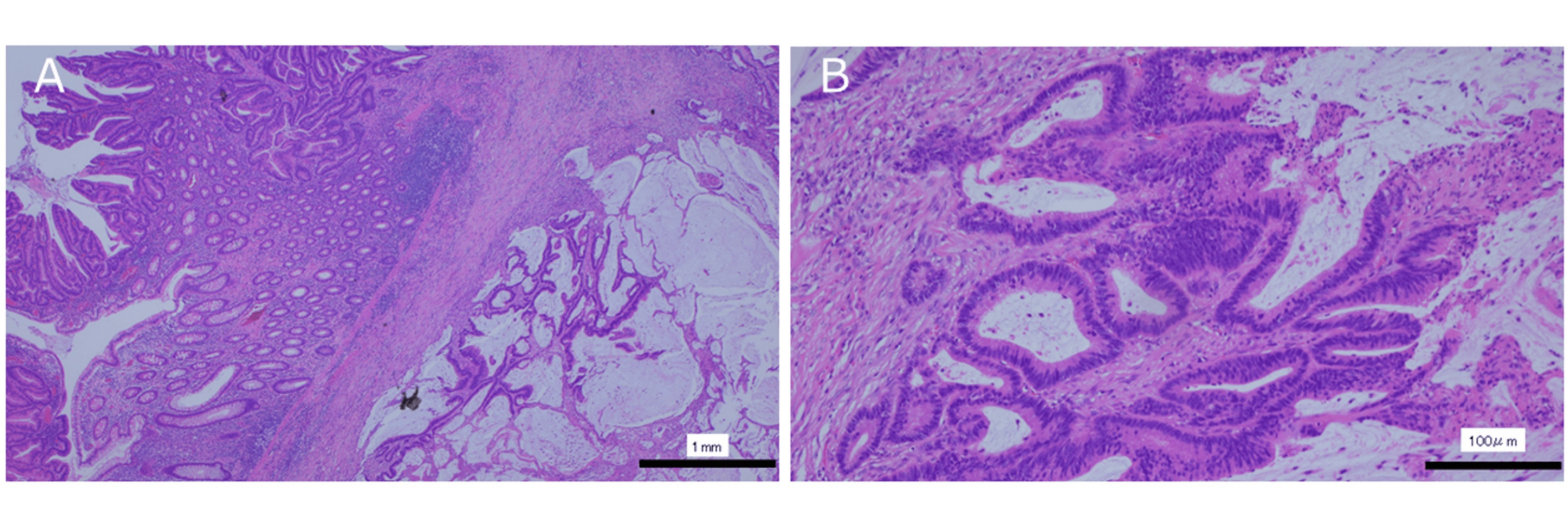
Histopathological findings Histopathological examination showed well- to moderately differentiated adenocarcinoma with focal mucinous components and subserosal invasion. (A) Low-power view and hematoxylin and eosin staining; (B) High-power view and hematoxylin and eosin staining

Histopathological examination revealed a predominantly well- to moderately differentiated adenocarcinoma with focal mucinous components (Figures 6A, 6B).

The tumor invaded the subserosal layer but showed no serosal surface exposure. The distances from the tumor to the proximal and distal resection margins were 9.0 cm and 8.5 cm, respectively, and all resection margins were negative. No lymph node metastases were identified in 26 harvested lymph nodes (0/26). The final pathological stage was pT3N0M0, stage IIA, according to the eighth edition of the AJCC/UICC TNM classification.

Perioperative immunosuppressive therapy was administered in consultation with transplant surgeons. Oral tacrolimus was continued until the day of surgery, administered intravenously on postoperative day 1, and resumed orally on postoperative day 2. Everolimus was withheld from the day of surgery and resumed on postoperative day eight. Methylprednisolone was switched to intravenous administration on the day of surgery and postoperative day 1 and resumed orally on postoperative day 2. No postoperative rejection or graft dysfunction was observed. The patient’s postoperative course was uneventful, with no surgical complications. Serum creatinine was 1.48 mg/dL (preoperative), 1.63 mg/dL and 1.18 mg/dL on postoperative days 1 and 7, respectively. Oral intake resumed on postoperative day 2, and the patient was discharged on postoperative day 9. No recurrence was observed during the three-month follow-up period.

## Discussion

In the present case, robot-assisted right hemicolectomy was safely performed in a kidney transplant recipient with ascending colon cancer located adjacent to an ipsilateral renal allograft. The main technical challenge was avoiding injury to the renal allograft and transplanted ureter while maintaining an oncologically adequate resection. Preoperative non-contrast CT allowed the assessment of the anatomical relationships among the tumor, renal allograft, and transplanted ureter. On the basis of these findings, a modified port configuration was planned to avoid the renal allograft and to account for the expected course of the transplanted ureter. The Arm 1 port was shifted 2 cm medially from its usual position and placed near the midline, while a 5-mm assistant port was placed in the left lateral abdomen. Adequate robotic-arm clearance and instrument mobility were confirmed after docking and before dissection. As no arm collisions or instrument limitations were observed, the procedure was continued robotically.

A key transplant-specific technical concern was the caudal and lateral mobilization of the ileocecal region adjacent to the renal allograft. During this step, traction with the Arm 1 instrument could have resulted in contact or compression of the allograft. To reduce this risk, wrist articulation was performed under direct visualization to obtain an appropriate traction angle, and unnecessary manipulation near the allograft was avoided. This step requires care to avoid excessive caudal or lateral dissection, which can bring the operative plane close to the renal allograft and the expected course of the transplanted ureter. The transplanted ureter was not exposed or manipulated intraoperatively. Instead, unnecessary deep caudal dissection near the expected ureteral course was avoided.

Kidney transplant recipients have an increased cumulative risk of malignancy over time, partly because of long-term immunosuppressive therapy and prolonged post-transplant survival [1]. Previous studies have suggested that these patients have a higher risk of developing advanced colorectal neoplasia and colorectal cancer than the general population [2,3]. Colorectal surgery in kidney transplant recipients requires careful attention to infectious complications, wound-related complications, acute kidney injury, and the perioperative adjustment of immunosuppressive therapy [5,6]. In the present case, immunosuppressive therapy was administered in consultation with transplant surgeons, and no surgical complications, rejection, or graft dysfunction were observed postoperatively.

Minimally invasive surgery is an established approach for selected patients with colorectal cancer [4,7]. However, the feasibility of minimally invasive surgery in kidney transplant recipients may depend on patient-specific anatomy, including the location of the renal allograft and the expected course of the transplanted ureter. Robot-assisted surgery may be useful in anatomically complex cases because the magnified three-dimensional view and wristed instruments can facilitate precise traction and dissection when port placement is constrained [8]. In this case, wristed articulation helped obtain an appropriate traction angle during mobilization near the renal allograft. The independent arm design of the Hugo™ RAS system may have facilitated flexible port placement and arm positioning. However, this single case could not determine whether this platform was superior to other robotic systems or conventional laparoscopy.

These transplant-specific precautions did not compromise oncological procedures. Despite modified port placement, adequate right colonic mobilization and complete mesocolic excision with central lymphadenectomy were achieved. The retrieval of 26 lymph nodes and negative resection margins supported the oncological adequacy of the resection.

This case report has several limitations. First, it describes a single case with a short postoperative follow-up period. Therefore, long-term oncological outcomes and changes in graft function cannot be adequately assessed. Second, this case report did not directly compare robot-assisted surgery with open surgery, conventional laparoscopy, or other robotic platforms. Further research is needed to clarify the generalizability of this approach in similar patients.

## Conclusions

Robot-assisted right hemicolectomy was performed without conversion in a kidney transplant recipient with ascending colon cancer adjacent to an ipsilateral renal allograft. This case suggests that careful preoperative anatomical assessment, individualized port placement, and confirmation of robotic arm clearance may help achieve safe, oncologically adequate, minimally invasive colectomy in selected kidney transplant recipients. Further accumulation of similar cases is needed to clarify the generalizability of this approach.
